# Healthcare-Associated Infections' Characteristics Among Burn Patients and Risk Factors of Mortality: A Study Based on Data From a Tertiary Center in Iran: Nosocomial Infections Among Burn Patients

**DOI:** 10.1155/2024/8707245

**Published:** 2024-10-28

**Authors:** Nima Mozafari, Effat Abbasi Montazeri, Sasan Moogahi, Seyed Mohammad Amin Alavi

**Affiliations:** ^1^Faculty of Medicine, Ahvaz Jundishapur University of Medical Sciences, Ahvaz, Iran; ^2^Infectious and Tropical Diseases Research Center, Health Research Institute, Ahvaz Jundishapur University of Medical Sciences, Ahvaz, Iran; ^3^Department of Microbiology, Faculty of Medicine, Ahvaz Jundishapur University of Medical Sciences, Ahvaz, Iran

**Keywords:** antimicrobial stewardship, burns, healthcare-associated infection, microbial sensitivity test, mortality

## Abstract

**Background:** Burn patients are more likely to get healthcare-associated infections (HAIs). The current study aimed to investigate the characteristics of HAI and mortality risk factors among burn patients admitted to a tertiary center in Iran.

**Methods:** A retrospective study was conducted in 2021 on burn patients who developed HAI after hospitalization in a tertiary center in Ahvaz, Iran. The records of patients admitted and managed between March 2019 and March 2020 were reviewed. Statistical analysis was carried out using IBM SPSS Version 26, with *p* < 0.05 considered statistically significant.

**Results:** Of the 1,659 admitted patients, 395 (23.8%) developed HAI during the study period. After excluding patients with incomplete medical records and those with fungal or viral infections, 363 patients remained. The majority of these cases occurred in male patients. The most common type of HAI was burn wound infection (56.2%). Patients were mainly affected by Gram-negative bacteria, *Pseudomonas aeruginosa* (39.7%), while the most common Gram-positive bacteria was *Staphylococcus epidermididis* (9.6%). The most common antibiotic resistance among *P. aeruginosa*-infected patients was reported against imipenem, followed by gentamicin and ciprofloxacin; however, the mentioned organism was mainly sensitive to colistin. Gender, age, bloodstream infection (BSI), ventilator-associated infection (VAI), ICU admission, and total burned surface area (TBSA) resulted in 3.585, 1.028, 2.222, 7.469, 5.278-, and 1.031 times higher mortality rates, respectively.

**Conclusion:** Female gender, advanced age, BSI, VAI, and ICU admission are risk factors for HAI. These findings emphasize the need for focused infection prevention and management to improve high-risk burn patient survival.

## 1. Introduction

Burns are described in the literature as a form of injury resulting from inadvertent contact with substances of elevated temperature, including hot liquids, solids, or gases such as cooking stoves, smoke, steam, drinks, equipment, devices, instruments, radiators, and heat-emitting objects [1]. James et al. pointed out that the global prevalence of burns in 2017 was reported as 209 million cases, and the incidence of burns was 14 million cases. The age-standardized incidence rate of burns decreased from 275 per 100,000 person-years in 1990 to 215 per 100,000 person-years in 2019. The World Health Organization (WHO) reported 180,000 deaths due to burns annually, mainly in low- and middle-income countries. During the past decades, the mortality rate following burn injury has steadily decreased due to advances in burn wound care and resuscitation techniques and a decrease in the average severity of burn injuries [2–5].

Patients with burn injuries are more susceptible to infection due to an impaired host defense resulting from a damaged skin barrier; moreover, nosocomial infections are more prevalent among burn patients than other patients [6, 7]. Common infections observed in individuals with burn injuries include cellulitis, burn wound infection (BWI), pneumonia, urinary tract infection (UTI), bloodstream infection (BSI), and infection of the venous access, whether central or peripheral [8].

Opportunistic bacteria, including *Enterococcus faecalis*, *Staphylococcus aureus*, *Klebsiella pneumoniae*, *Acinetobacter baumannii*, *Pseudomonas aeruginosa*, and *Enterobacter* spp. are major causes of infection in burn victims [9]. Furthermore, antibiotic resistance is the primary obstacle when treating bacterial infections, and it has been highly reported for *P. aeruginosa*, *S. aureus*, and *A. baumannii* [10].

Infections are the most common cause of death among burn victims after initial resuscitation [11]. The literature indicates a range of parameters that are associated with death in patients with severe burns while being treated in the critical care burn unit. The factors that are believed to elevate death rates significantly include age, gender, total burned surface area (TBSA), the presence of third-degree burns, and inhalation injury [12].

The current study aims to investigate the characteristics of healthcare-associated infections (HAIs) and mortality risk factors among burn patients admitted to a tertiary center in Iran. Previous studies have primarily focused on the microbiological profiles of HAI among burn patients. Fewer studies have investigated the risk factors associated with mortality in patients who develop HAI after being admitted to the hospital for burn management. To the best of our knowledge, the current study is a forerunner in investigating the region's aforementioned characteristics and risk factors. We utilized a retrospective design which analyzes medical record data to assess patterns and associations between different types of HAI and mortality risk factors in burn patients.

## 2. Methods

### 2.1. Study Population

A retrospective study was conducted in May 2020 on burn patients who developed HAI after hospitalization in Taleghani Hospital, affiliated with Ahvaz Jundishapur University of Medical Sciences, where the majority of burn patients from the Southwest of Iran were referred for further investigations. The records of patients admitted and managed between March 2019 and March 2020 were reviewed. The inclusion criteria were as follows: (a) development of HAI after hospitalization, (b) presence of bacterial infection based on wound, blood, urine, nasopharyngeal swab, or endotracheal tube cultures, and (c) antibiogram test results. The exclusion criteria were (a) incomplete medical records and (b) other infections, including viral or fungal infections. The inclusion and exclusion criteria were chosen to focus on bacterial HAI, which are most relevant for studying infection control in burn patients. This focus aims to assist physicians in more effectively managing such infections if they occur. A disk diffusion method was applied to carry out the antibiogram.

### 2.2. Ethics Approval

The Ethics Committee of Ahvaz Jundishapur University of Medical Sciences approved the study design (Ethics code: ir.ajums.medicine.rec.1398.024). The retrospective design of the study resulted in the Institutional Ethics Committee of Ahvaz Jundishapur University of Medical Sciences waiving the written informed consent from all individuals. All methods were carried out in accordance with the Declaration of Helsinki.

### 2.3. HAIs

Based on the literature, HAIs refer to a collection of infections that a patient does not possess prior to their admission into a healthcare facility or even in the latency period. The aforementioned infections manifest themselves either upon initial arrival at the medical facility or 48–72 h after admission [13]. The diagnosis of HAI in burn patients was made by a multidisciplinary team of physicians, including an infectious disease specialist, a surgeon, an internal medicine specialist, and an intensivist.

### 2.4. Data Specifications

Data included demographic characteristics (age and gender), type of HAI, type of bacteria based on gram stain, designated ward (burn unit or burn intensive care units [ICUs]), duration of hospitalization, patients' outcome (alive or expired), TBSA (the surface body area affected by burns), presence of secondary blood infection, and antibiotic susceptibility or resistance.

### 2.5. Statistical Analysis

All patients were anonymized and given identification codes. Statistical analysis was carried out using IBM SPSS Version 26 (IBM Corp., Armonk, NY, USA). The mean and standard deviation (SD) for qualitative variables and the number and percentage for quantitative variables were expressed. To further investigate the characteristics of patients who either fully recovered from HAI or died and to identify the risk factors associated with mortality, patients were categorized into two groups: (a) the survivors group and (b) the deceased group. A Chi-square test and Fisher's exact tests were used to compare the categorical variables, and the distribution of quantitative variables was examined for normality using the Shapiro–Wilk test. Parametric tests, such as the independent *t*-test, were utilized to compare the variables with normal distribution. The Mann–Whitney *U* test was further utilized to compare variables that did not have a normal distribution. In addition, binary logistic regression analysis was performed to assess the effect of age, gender, and all other relevant factors. *p* < 0.05 was considered statistically significant.

## 3. Results

One-thousand six-hundred fifty nine burn patients were admitted to Taleghani Hospital during the study period, of which 395 (23.8%) developed HAI. Twelve patients were excluded due to incomplete medical records, and an additional 20 were excluded because of other infections, including viral or fungal infections. A complete list of demographic and clinical characteristics of the patients is described in Table 1. A statistically significant difference in terms of gender among both groups was observed (*p* < 0.001). The mean age of the alive and expired groups was 31.0 ± 21.56 years and 43.4 ± 21.83 years, respectively, which in itself shows a statistically significant difference among both groups (*p* < 0.001). The most prevalent type of HAI is BWI (56.2%), followed by BSI (20.9%, for which there was a statistically significant difference among both groups (*p* < 0.001). A total of 81% of HAI were caused by Gram-negative bacteria during the study period. There was no statistically significant difference among both groups regarding the type of bacteria based on gram stain and secondary bacterial infection (*p*=0.123 and *p*=0.765, respectively). Two hundred and thirty four (64.5%) patients were admitted to the ICU, the majority of whom expired (*p* < 0.001). There were also statistically significant differences in terms of TBSA and duration of hospitalization (*p* < 0.001 and *p*=0.049, respectively).

A complete list of bacterial pathogens causing different HAI is described in Table 2. The most prevalent Gram-negative bacteria causing HAI was reported as being *P. aeruginosa* (49%), followed by *Escherichia coli* (28.2%), whereas in the present investigation, the most prevalent Gram-positive bacteria causing HAI was *Staphylococcus epidermidis* (50.7%), followed by *S. aureus* (37.7%). *P. aeruginosa* was also the leading cause of all types of HAI, except for UTI, for which the leading cause was *E. coli*.

Antibiotic susceptibility and resistance of different bacterial pathogens are summarized in Table 3. The highest number of antibiotic susceptibilities of *P. aeruginosa* was observed with colistin (117 cases), while the highest number of resistances was reported with imipenem (78 cases). *E. coli* showed the highest susceptibility to imipenem and ciprofloxacin, with both antibiotics effective in 31 cases. In addition, *S. epidermidis*-infected patients exhibited the greatest response to clindamycin (10 cases) and vancomycin (25 cases).

A multivariable binary logistic regression was utilized to control the effect of the associated factors, including gender, age, HAI type, type of bacteria based on Gram stain, designated ward, duration of hospitalization, and TBSA. The results of the multivariable binary logistic regression (Table 4) revealed that gender, age, HAI type, designated ward, and TBSA had statistically significant associations with patients' outcomes (*p* < 0.05). However, other variables, including the type of bacteria based on Gram stain and duration of hospitalization, showed no statistically significant association (*p* > 0.05). Female burn patients were 3.5 times (OR = 3.585, 95% CI: 2.097–6.129, and *p* < 0.001) more likely to die of HAI than males. The mortality rates of burn patients admitted to the ICU were 5.3 times higher (OR = 5.278, 95% CI: 2.741–10.163, and *p* < 0.001) than those admitted to the burn wards. Among the different HAIs, only BSI and VAI showed a statistically significant association, of which the BSI and VAI patients had 2.2- (OR = 2.222, 95% CI: 1.157–4.266, and *p*=0.016) and 7.5 (OR = 7.469, 95% CI: 1.240–44.969, and *p*=0.028) times higher mortality rates compared to BWI, respectively. Furthermore, an increase of 1 year in tandem with 1% TBSA increased the likelihood of burn patients dying of HAI.

## 4. Discussion

The current study's HAI rate was nearly 24%, and the mortality rate was 35.5%. Gram-negative bacteria, among which *P. aeruginosa* and *E. coli* were the most common pathogens, were the cause of most of the HAI. Moreover, the most common Gram-positive bacteria were *S. epidermidis* and *S. aureus*. There was an association between gender, age, HAI type, designated ward, and TBSA with the mortality rate of burn patients compromised by HAI. Female gender, BSI, and VAI were associated with 3.585, 2.222- and 7.469 times higher mortality rates compared to male patients and BWI, respectively.

Öncül et al., in a study in Turkey, reported that more than 70% of burn patients admitted to ICU developed HAI [14]. Due to the inclusion of only ICU-admitted burn patients, the reported rate of HAI was much higher compared to the current study. In another study by Escandón-Vargas et al., the rate of HAI among burn patients was almost 29% and the mortality rate was 4.3% [15]. The reason for the difference between the two studies may be due to the higher study population of the current investigation. Corcione et al. reported that the majority of the burn patients who developed infection were male [16], which was similar to the current investigation.

Based on Wang et al., the most prevalent type of HAI was BWI, followed by BSI [17]. Another study by Benchamkha et al. also reported that the most common bacterial infection site among burn patients was BWI, followed by BSI and UTI [18]. The result of the abovementioned studies parallels that of the current study. Gong et al. reported that the majority of infections occur in burn wards [19]. However, HAI is mainly reported in the ICU in the current investigation, which may be due to a lower study population compared to the aforementioned study.

Zhang et al. reported that the majority of pathogens isolated from thermal burn patients were Gram-negative [20, 21]. Gupta et al. have pointed out that *P. aeruginosa* is the primary etiology of infection in burn patients [22, 23], which parallels the results of the current study. However, other studies found *S. aureus* as the most common pathogen among burn patients who developed an infection [24, 25]. It is believed that the variation in etiological organisms could be attributed to the number of participants and geographical differences. In a study conducted in Turkey, Öncül et al. reported that *P. aeruginosa* was the primary pathogen responsible for HAI in patients with BWI, BSI, and UTI. Conversely, *Acinetobacter baumannii* was identified as a leading etiological agent for pneumonia [14]. The variation in predominant pathogens may be attributed to differing infection prevention protocols employed in the hospitals. In addition, the discrepancy could be related to the fact that Öncül et al. exclusively studied patients in burn ICUs, while patients in general wards were not included in their research.

Pujji et al. found that the most sensitive antibiotic to Gram-negative bacteria was colistin, and the most sensitive antibiotic to Gram-positive bacteria was vancomycin [26]. The said results paralleled the results of the current study. Another investigation by Singh et al. investigated the evolving patterns of antibiotic susceptibility among bacterial isolates in a burn unit, in which it was seen that *P. aeruginosa* was resistant primarily against ceftazidime, followed by gentamicin and ciprofloxacin [27]. In a study conducted by Gupta, Naik, and Singh, colistin and tigecycline were reported to exhibit the lowest relative frequency of antibiotic resistance among *E. coli*, *Proteus mirabilis*, *Burkholderia cepacia*, *Pseudomonas species*, *K*. *pneumoniae*, *A*. *baumannii*, *and Enterobacter cloacae* [22]. The discrepancy might be because both studies only evaluated wound samples. Furthermore, Bourgi et al. reported that in burn patients infected with *P. aeruginosa*, linezolid was noted to have the lowest frequency of antibiotic resistance. However, no difference between colistin and linezolid was reported in patients infected with *E. coli* [28]. In another study by Öncül et al., meropenem was found to be highly effective against Gram-negative infections, while vancomycin, teicoplanin, and linezolid showed high susceptibility against Gram-positive infections [14].

Öncül et al. found that TBSA > 40%, full-thickness burn, age > 65, and inhalation injury were independent risk factors for nosocomial infections [14]. However, the mechanisms and degree of the burn injury were not studied in the current investigation. According to Escandón-Vargas et al., several risk factors were associated with an increased likelihood of HAI, including mestizo ethnicity, flame burns, scald burns, burns covering 21%–40% TBSA, burns exceeding 40% TBSA, and the use of central venous catheters [15]. Another investigation by Alp et al. studied the risk factors for nosocomial infection and mortality, in which the authors found an association between advanced age, flame injury, high TBSA, admission after 24 h of burn injury, having an underlying disease, and nosocomial infection with mortality [29]. The aforementioned study evaluated risk factors for mortality of burn patients with or without nosocomial infection; however, the mechanism of the burn injury and underlying diseases were not determined in the current study. Chen et al. investigated incidence and mortality among individuals admitted to hospitals with moderate to severe burn injuries, in which BSI, ICU admission, and TBSA with full-thickness injuries ≥ 50% were prognostic factors of mortality [30]. The results obtained paralleled the findings of the current study. Guo et al.'s investigation of 156 HAI patients estimated the impact of nosocomial infection on mortality in burn patients and reported that nosocomial infection, TBSA, and inhalation injury were associated with death among burn patients; however, gender and age were not associated with death [31]. The reason for the differences between Guo et al.'s study and the results of the current study might be due to the sample size, in which the current study investigated 363 HAIs among burn patients. In another study by Hu et al., acute kidney injury (AKI) and septic shock were identified as independent predictors of 30-day mortality in burn patients with BSI [32]; however, Hu et al. focused exclusively on burn patients with BSI and did not investigate other types of infections among burn patients.

## 5. Conclusion

The study underscores the high prevalence and significant mortality associated with HAI among burn patients, identifying factors such as female gender, advanced age, BSI, VAI, and ICU admission as key predictors of poor outcomes. *P. aeruginosa* emerged as the most common pathogen, with antibiotic resistance posing a major challenge in treatment. These findings highlight the critical need for targeted infection control and management strategies to improve survival rates among high-risk burn patients.

### 5.1. Limitations

The number of participants and the short-term length of the study were the most crucial limitations of the current investigation. Another critical limitation was that the authors did not include the mechanism of burn injury, grade of infection, and underlying diseases in the current study, which the authors highly suggest including in future studies.

## Figures and Tables

**Table 1:** Demographic and clinical characteristics of burn patients who developed healthcare-associated infections (HAIs).

Feature		Total (*n* = 363)	Alive group (*n* = 234)	Expired group (*n* = 129)	*p*
Gender (male)		230 (63.4%)	171 (73.1%)	59 (45.7%)	< 0.001

Age, mean (st. d.), years		35.4 ± 22.426	31 ± 21.559	43.37 ± 21.835	< 0.001

Infection type	BWI	204 (56.2%)	149 (63.7%)	55 (42.6%)	< 0.001
	BSI	76 (20.9%)	39 (16.7%)	37 (28.7%)	
	UTI	64 (17.6%)	40 (17.1%)	24 (18.6%)	
	VAI	11 (3%)	2 (0.9%)	9 (7%)	
	Pneumonia	8 (2.2%)	4 (1.7%)	4 (3.1%)	

Type of bacteria based on Gram stain	Gram-positive	69 (19%)	50 (21.4%)	19 (14.7%)	0.123
	Gram-negative	294 (81%)	184 (78.6%)	110 (85.3%)	

Designated ward	Burn ward	129 (35.5%)	113 (48.3%)	16 (12.4%)	< 0.001
	Burn ICU	234 (64.5%)	121 (51.7%)	113 (87.6%)	

Secondary blood infection		10 (2.8%)	6 (2.6%)	4 (3.1%)	0.765

TBSA		41.17 ± 16.141	38.3 ± 15.580	46.37 ± 15.893	< 0.001

Duration of hospitalization		35.82 ± 73.570	38.52 ± 78.202	30.92 ± 64.323	0.049

Abbreviations: BSI, bloodstream infection; BWI, burn wound infection; ICU, intensive care unit; TBSA, total body surface area; UTI, urinary tract infection; VAI, ventilator-associated infection.

**Table 2:** Complete description of organisms and types of healthcare-associated infection among burn patients.

Organism	Type of infection				
	BWI (*n* = 204)	BSI (*n* = 76)	UTI (*n* = 64)	VAI (*n* = 11)	Pneumonia (*n* = 8)
*P. aerigenosa*	93 (45.6%)	32 (42.1%)	11 (17.2%)	3 (27.3%)	5 (62.5%)
*E. coli*	34 (16.7%)	12 (15.8%)	35 (54.7%)	1 (9.1%)	1 (12.5%)
*Enterobacter*	24 (11.8%)	2 (2.6%)	6 (9.4%)	2 (18.2%)	1 (12.5%)
*S. epidermidis*	15 (7.4%)	12 (15.8%)	5 (7.8%)	2 (18.2%)	1 (12.5%)
*Acinetobacter*	16 (7.8%)	8 (10.5%)	0	2 (18.2%)	0
*S. aureus*	14 (6.9%)	9 (11.8%)	2 (3.1%)	1 (9.1%)	0
*S. saprophyticus*	1 (0.5%)	1 (1.3%)	3 (4.7%)	0	0
*Proteus*	3 (1.5%)	0	0	0	0
*Citrobacter*	2 (1%)	0	0	0	0
*Enterococci*	1 (0.5%)	0	1 (1.6%)	0	0
*klebsiella*	0	0	1 (1.6%)	0	0
*S. viridans*	1 (0.5%)	0	0	0	0

Abbreviations: BSI, bloodstream infection; BWI, burn wound infection; UTI, urinary tract infection; VAI, ventilator-associated infection.

**Table 3:** Complete description of organisms and antibiotic susceptibility and resistance.

Type of antibiotic	Type of bacteria													
	Resistance	*P. aerigenosa*	*E. coli*	*Enterobacter*	*S. epidermidis*	*Acinetobacter*	*S. aureus*	*S. saprophyticus*	*Proteus*	*Citrobacter*	*Enterococci*	*klebsiella*	*viridans streptococci*	Total
Erythromycin	R	0	0	0	2	0	0	0	0	0	0	0	0	2
	S	0	0	0	0	0	0	0	0	0	0	0	0	0
	Semi	0	0	0	0	0	0	0	0	0	0	0	0	0

Oxacillin	R	1	0	1	6	0	6	2	0	0	0	0	0	16
	S	0	1	0	1	0	6	1	0	0	0	0	0	9
	Semi	0	0	0	0	0	0	1	0	0	0	0	0	1

Ampicillin	R	1	0	0	1	0	0	0	0	0	0	0	0	2
	S	0	1	0	0	0	0	0	0	0	0	0	0	1
	Semi	0	0	0	0	0	0	0	0	0	0	0	0	0

Amoxicillin	R	0	0	0	0	1	0	0	0	0	0	0	0	1
	S	0	0	0	0	0	0	0	0	0	0	0	0	0
	Semi	0	0	0	0	0	0	0	0	0	0	0	0	0

Amikacin	R	48	20	9	6	12	2	0	0	0	0	0	1	98
	S	12	7	2	4	0	4	1	0	0	0	0	0	30
	Semi	1	0	0	0	0	0	0	0	0	0	0	0	1

Imipenem	R	78	31	14	1	19	0	0	1	1	0	0	0	145
	S	9	7	0	1	0	0	1	0	1	1	1	0	21
	Semi	1	1	0	0	0	0	0	0	0	0	0	0	2

Piperacillin	R	37	15	2	3	1	0	0	2	0	0	0	0	60
	S	7	3	0	0	0	0	0	1	0	0	0	0	11
	Semi	0	0	0	0	0	0	0	0	0	0	0	0	0

Piperacillin/tazobactam	R	1	0	0	0	0	0	0	0	0	0	0	0	1
	S	0	0	0	0	0	0	0	0	0	0	0	0	0
	Semi	0	0	0	0	0	0	0	0	0	0	0	0	0

Gemifloxacin	R	0	0	0	0	1	0	0	0	0	0	0	0	1
	S	0	0	0	0	0	0	0	0	0	0	0	0	0
	Semi	0	0	0	0	0	0	0	0	0	0	0	0	0

Gentamicin	R	73	28	14	4	15	2	0	2	0	2	0	0	140
	S	2	18	4	2	0	4	1	0	0	0	1	0	32
	Semi	0	0	0	0	0	0	0	0	0	0	0	0	0

Cefazolin	R	3	9	4	1	1	1	1	0	0	0	1	0	21
	S	0	2	1	0	0	0	0	0	0	0	0	0	3
	Semi	0	0	0	0	0	0	0	0	0	0	0	0	0

Cefepime	R	1	1	0	0	0	0	0	0	0	0	0	0	2
	S	0	0	0	0	0	0	0	0	0	0	0	0	0
	Semi	0	0	0	0	0	0	0	0	0	0	0	0	0

Ceftazidime	R	64	22	13	2	19	1	1	2	1	0	0	0	125
	S	14	10	2	0	0	1	0	1	0	0	0	0	28
	Semi	1	0	0	0	0	0	0	0	0	0	0	0	1

Ceftriaxone	R	2	6	9	1	2	0	1	0	0	0	0	0	21
	S	0	0	0	0	0	1	1	0	0	0	0	0	2
	Semi	0	0	0	0	0	0	0	0	0	0	0	0	0

Cefixime	R	0	1	0	0	0	0	0	0	0	0	0	0	1
	S	0	0	0	0	0	0	0	0	0	0	0	0	0
	Semi	0	0	0	0	0	0	0	0	0	0	0	0	0

Cotrimoxazole	R	2	1	0	1	1	2	0	0	0	0	0	0	7
	S	0	0	0	0	0	1	0	0	0	0	0	0	1
	Semi	0	0	0	0	0	0	0	0	0	0	0	0	0

Flucloxacillin	R	5	2	0	8	0	5	2	0	0	0	0	1	23
	S	0	0	0	11	0	9	1	0	0	0	0	0	21
	Semi	0	0	0	0	0	0	0	0	0	0	0	0	0

Ciprofloxacin	R	71	31	22	6	15	7	1	1	1	1	0	1	157
	S	9	14	7	9	0	3	1	0	1	1	1	0	46
	Semi	0	1	0	0	0	0	0	0	0	0	0	0	1

Colistin	R	5	4	0	2	0	2	1	2	0	1	0	0	17
	S	117	35	23	0	22	1	1	0	2	0	0	0	201
	Semi	0	0	0	0	0	0	0	0	0	0	0	0	0

Clindamycin	R	2	2		10		5	0	0	0	0	0	0	19
	S	5	3	3	7	2	6	0	0	0	0	0	0	26
	Semi	0	0	0	0	0	0	1	0	0	0	0	0	1

Sulfamethoxazole	R	35	27	15	9	2	3	2	1	0	0	0	1	95
	S	8	9	6	4	1	8	0	0	0	0	0	0	36
	Semi	0	0	0	1	0	0	0	0	0	1	0	0	2

Levofloxacin	R	3	5	3	1	1	0	0	0	1	0	0	0	14
	S	0	1	0	0	0	0	0	0	0	0	0	0	1
	Semi	0	0	0	0	0	0	0	0	0	0	0	0	0

Meropenem	R	22	12	5	0	1	0	0	0	0	0	0	0	40
	S	1	3	0	0	0	0	0	0	0	0	0	0	4
	Semi	0	0	0	0	0	0	0	0	0	0	0	0	0

Nitrofurantoin	R	2	7	2	1	0	1	0	0	0	1	0	0	14
	S	0	17	3	5	0	2	0	0	0	1	0	0	28
	Semi	0	0	0	0	0	0	0	0	0	0	0	0	0

Vancomycin	R	0	0	0	2	0	0	1	0	0	2	0	1	6
	S	5	0	0	25	0	19	2	0	0	0	0	0	51
	Semi	0	0	0	0	0	0	1	0	0	0	0	0	1

Abbreviations: R, resistance; S, susceptibility; Semi, semiresistance.

**Table 4:** Multivariable binary logistic regression revealing risk factors of mortality.

Variable	Odds ratio (OR)	Confidence interval (CI)		*p*
		Lower	Upper	
Gender (female)	3.585	2.097	6.129	< 0.001
Age	1.028	1.016	1.041	< 0.001
Urinary tract infection (UTI)	1.344	0.661	2.734	0.415
Blood stream infection (BSI)	2.222	1.157	4.266	0.016
Ventilator-associated infections (VAIs)	7.469	1.240	44.969	0.028
Pneumonia	2.163	0.371	12.626	0.391
Type of bacteria based on Gram stain	1.773	0.859	3.663	0.122
Designated ward (burn ICU)	5.278	2.741	10.163	< 0.001
Duration of hospitalization	1	0.996	1.004	0.921
Total body surface area (TBSA)	1.031	1.014	1.049	< 0.001

## Data Availability

The data used to support the findings of this study are available from the corresponding author upon reasonable request.
